# Supplementary material for: Evaluation of the Recipe Function in Popular Dietary Smartphone Applications, with Emphasize on Features Relevant for Nutrition Assessment in Large-Scale Studies
Source: Nutrients. 2019 Jan 19;11(1):200. doi: 10.3390/nu11010200 (PMC6356331; doi:10.3390/nu11010200)
Supplement: Supplementary file 1 [file nutrients-11-00200-s001.zip › Supplementary Macronutrients.pdf]

Table S1: Energy and macronutrient differences between app and NEVO for stamppot

	Fat (g)	Protein (g)	Carbohydrate (g)	Energy (kcal)	Energy difference with reference	Fat difference with reference	Protein difference with reference	Carbohydrate difference with reference
Reference raw	10.9	16.95	70.375	472.25	0	0	0	0
Myfitnesspal	10.7	17	70.3	476	3.75	-0.2	0.05	-0.075
Fatsecret	5.78	17.39	70.66	430	-42.25	-5.12	0.44	0.285
YAZIO	10.5	17.7	82.2	482	9.75	-0.4	0.75	11.825
Lose it!	7.2	17.8	71.6	456	-16.25	-3.7	0.85	1.225
Lifesum	9.9	11.8	67.5	403	-69.25	-1	-5.15	-2.875
MyPlate	10.33	15.26	80.53	444	-28.25	-0.57	-1.69	10.155
MyNetDiary	10.75	16.75	55.25	419	-53.25	-0.15	-0.2	-15.125
Calories!	10	11.7	56.3	379.6	-92.65	-0.9	-5.25	-14.075
The Secret of Weight		-	-	356.25	-116	-	-	-
Virtuagym Food	11.8	15.03	59.02	410	-62.25	0.9	-1.92	-11.355
Nutracheck	17.5	5.9	61.4	531	58.75	6.6	-11.05	-8.975
HI	8	0	64.3	428.1	-44.15	-2.9	-16.95	-6.075

Table S2: Energy and macronutrient differences between app and NEVO for pizza salami, tomato, mushroom

	Fat (g)	Protein (g)	Carbohydrate (g)	Energy (kcal)	Energy difference with reference	Fat difference wit reference	Protein difference with reference	Carbohydrate difference with reference
Reference raw	25.925	22.075	38.8	482.75	0	0	0	0
Myfitnesspal	23.3	19.8	38.9	447	-35.75	-2.625	-2.275	0.1
Fatsecret	25.64	20.87	39.41	478	-4.75	-0.285	-1.205	0.61
YAZIO	26.2	21.9	39.1	481	-1.75	0.275	-0.175	0.3
Lose it!	23	19.4	40.7	440.4	-42.35	-2.925	-2.675	1.9
Lifesum	25.6	21.3	40.7	478	-4.75	-0.325	-0.775	1.9
MyPlate	21.48	19.49	50.63	448	-34.75	-4.445	-2.585	11.83
MyNetDiary	25.25	17	38	482.75	0	-0.675	-5.075	-0.8
Calories!	24.3	21.1	36	459.2	-23.55	-1.625	-0.975	-2.8
The Secret of Weight	-	-	-	476	-6.75	-	-	-
Virtuagym Food	25.85	21.13	38.42	475	-7.75	-0.075	-0.945	-0.38
Nutracheck	20.5	19.5	43	436	-46.75	-5.425	-2.575	4.2
HI	23	18.3	36	442.1	-40.65	-2.925	-3.775	-2.8

Table S3: Energy and macronutrient differences between app and NEVO for hachee

	Fat (g)	Protein (g)	Carbohydrate (g)	Energy (kcal)	Energy difference with reference	Fat difference with reference	Protein difference with reference	Carbohydrate difference with reference
Reference raw	17.85	23.325	13.7	315.5	0	0	0	0
Myfitnesspal	20	22.4	15.4	330	14.5	2.15	-0.925	1.7
Fatsecret	13.59	22.7	17.51	273	-42.5	-4.26	-0.625	3.81
YAZIO	13.4	22.3	17.4	269	-46.5	-4.45	-1.025	3.7
Lose it!	9.1	12.1	17.5	197	-118.5	-8.75	-11.225	3.8
Lifesum	20.3	22.5	12.8	322	6.5	2.45	-0.825	-0.9
MyPlate	19.51	2.05	15.95	327	11.5	1.66	-21.275	2.25
MyNetDiary	26.25	22	9	390.25	74.75	8.4	-1.325	-4.7
Calories!	17.6	35.8	9.6	347.5	32	-0.25	12.475	-4.1
The Secret of Weight	-	-	-	373	57.5	-	-	-
Virtuagym Food	12.72	24.59	13.17	270	-45.5	-5.13	1.265	-0.53
Nutracheck	28.6	32.3	16.8	457	141.5	10.75	8.975	3.1
HI	20.2	23.2	12	334.4	18.9	2.35	-0.125	-1.7
