# Supplementary material for: Evaluation of the Recipe Function in Popular Dietary Smartphone Applications, with Emphasize on Features Relevant for Nutrition Assessment in Large-Scale Studies
Source: Nutrients. 2019 Jan 19;11(1):200. doi: 10.3390/nu11010200 (PMC6356331; doi:10.3390/nu11010200)
Supplement: Supplementary file 1 [file nutrients-11-00200-s001.zip › Supplementary Micronutrients.pdf]

Table S4. Calcium calculation for three recipes

stamppot		Raw weight in recipe (g)	Ca mg in recipe(NEVO)	Ca mg in recipe (Mynetdiary)	Ca mg in recipe (Calories!)	Ca mg in recipe(Virtue)	First and second cooking procedure	First and second retention factor	Ca mg in one portion (NEVO)
	kale	250.00	450.00	377.50	530.00	337.50	stew	1.00	450.00
	potatoes	312.50	18.75	28.13	18.75	28.13	stew	1.00	18.75
	butter	10.00	1.70	2.40	1.30	2.40	boil	1.00	1.70
	semi-skimmed milk	18.80	23.12	22.94	23.12	23.31	boil	1.00	23.12
	Total		493.57	430.96	573.17	391.34			493.57
Pizza	Flour	50.00	7.50	7.50	7.80	7.50	bake in oven	1.00	7.50
	Olive oil	4.50	0.00	0.05	0.04	0.05	bake in oven	1.00	0.00
	Tomato puree	11.25	5.51	2.03	4.95	2.03	bake in oven	1.00	5.51
	Yeast	0.88	0.70	0.26	0.24	0.26	bake in oven	-	0.70
	Oregano	0.38	5.91	5.99	0.00	5.99	bake in oven	-	5.91
	Mature cheese 48+	37.50	306.00	270.38	270.00	270.38	bake in oven	1.00	306.00
	Tomato	25.00	2.75	2.50	2.50	2.50	bake in oven	1.00	2.75
	Salami	25.00	8.75	3.25	4.00	3.25	bake in oven	1.00	8.75
	Mushroom	25.00	1.50	0.75	0.75	0.75	bake in oven	1.00	1.50
	Total		338.62	292.70	290.28	292.70			338.62
Hachee	Hachee meat	100.00	5.00	13.00	4.00	14.00	fry in pan and stew	1.00 * 1.00	5.00
	Onion	100.00	29.00	23.00	23.00	23.00	fry in pan and stew	1.00 * 1.00	29.00
	Vinegar	4.50	0.14	1.22	0.72	1.35	stew	1.00	0.14
	Flour	7.50	0.75	1.13	1.20	1.13	stew	1.00	0.75
	Pepper	1.25	5.46	5.54	5.30	5.54	stew	1.00	5.46
	Butter	12.50	2.13	3.00	1.60	3.00	stew	1.00	2.13
	Laurel	1.25	0.00	10.43	0.00	10.43	stew	1.00	0.00
	Cloves	1.25	8.08	7.90	9.10	8.08	stew	1.00	8.08
	Bouillon powder	1.25	0.00	0.75	2.80	0.09	stew	1.00	0.00
	Total		50.55	65.95	47.72	66.60			50.55

Table S5. Vitamin C calculation for three recipes

		Raw weight in recipe (g)	VC mg in recipe(NEVO)	VC mg in recipe (Mynetdiary)	VC mg in recipe (Calories!)	VC mg in recipe(Virtue)	First and second cooking procedure	First and second retention factor	VC mg in one portion (NEVO)
stamppot	kale	250.00	250.00	299.00	252.50	300.00	stew	0.60	150.00
	potatoes	312.50	43.75	28.30	50.00	61.50	stew	0.85	37.19
	butter	10.00	0.00	0.00	0.00	0.00		-	0.00
	semi-skimmed milk	18.80	0.19	0.00	0.00	0.00	boil	0.70	0.13
	Total		293.94	327.30	302.50	361.50			187.19
Pizza	Flour	50.00	0.00	0.00	0.00	0.00	bake in oven	0.70	0.00
	Olive oil	4.50	0.00	0.00	0.00	0.00	bake in oven	-	0.00
	Tomato puree	11.25	1.46	1.24	4.19	1.19	bake in oven	0.80	1.17
	Yeast	0.88	0.00	0.00	0.00	0.00	bake in oven	-	0.00
	Oregano	0.38	0.00	0.00	0.00	0.01	bake in oven	-	0.00
	Mature cheese 48+	37.50	0.00	0.00	0.00	0.00	bake in oven	0.70	0.00
	Tomato	25.00	3.75	3.50	3.43	3.43	bake in oven	0.80	3.00
	Salami	25.00	0.00	0.00	0.00	0.00	bake in oven	0.80	0.00
	Mushroom	25.00	1.00	0.53	0.53	0.53	bake in oven	0.80	0.80
	Total		6.21	5.26	8.14	5.15			4.97
Hachee	Hachee meat	100.00	0.00	0.00	0.00	0.00	fry in pan and stew	0.75 * 2	0.00
	Onion	100.00	5.00	7.40	7.40	7.40	fry in pan and stew	0.85 * 2	3.61
	Vinegar	4.50	0.00	0.00	0.00	0.00	stew	1.00	0.00
	Flour	7.50	0.00	0.00	0.00	0.00	stew	1.00	0.00
	Pepper	1.25	0.00	0.00	0.00	0.00	stew	1.00	0.00
	Butter	12.50	0.00	0.00	0.03	0.03	stew	1.00	0.00
	Laurel	1.25	0.00	0.58	0.00	0.58	stew	1.00	0.00
	Cloves	1.25	1.01	0.00	0.00	1.01	stew	1.00	1.01
	Bouillon powder	1.25	0.00	0.00	0.00	0.01	stew	1.00	0.00
	Total		6.01	7.98	7.43	9.02			4.63

Table S6. Vitamin A calculation for three recipes

stamppot		Raw weight in recipe (g)	VA ug in recipe(NEVO)	VA ug in recipe (Mynetdiary)	VA ug in recipe (Calories!)	VA ug in recipe(Virtue)	First and second cooking procedure	First and second retention factor	VA ug in one portion (NEVO)
	kale	250.00	1677.50	2475.00	2250.00	0.00	stew	0.90	1509.75
	potatoes	312.50	3.13	7.50	0.00	0.00	stew	0.90	2.81
	butter	10.00	90.50	74.97	70.00	73.80		-	90.50
	semi-skimmed milk	18.80	3.20	0.00	0.00	0.00	boil	1.00	3.20
	Total		1774.32	2557.47	2320.00	73.80			1606.26
Pizza	Flour	50.00	0.00	0.35	0.00	0.00	bake in oven	0.90	0.00
	Olive oil	4.50	0.18	0.00	8.24	0.00	bake in oven	1.00	0.18
	Tomato puree	11.25	31.16	19.13	22.50	0.00	bake in oven	0.90	28.05
	Yeast	0.88	0.00	0.00	0.00	0.00	bake in oven	-	0.00
	Oregano	0.38	2.59	2.13	0.00	0.00	bake in oven	-	2.59
	Mature cheese 48+	37.50	129.38	125.25	136.13	96.75	bake in oven	1.00	129.38
	Tomato	25.00	19.25	58.25	37.48	0.00	bake in oven	0.90	17.33
	Salami	25.00	5.25	0.00	0.00	0.00	bake in oven	1.00	5.25
	Mushroom	25.00	0.00	0.00	0.00	0.00	bake in oven	1.00	0.00
	Total		187.81	205.10	204.34	96.75			182.76
Hachee	Hachee meat	100.00	20.00	1.98	34.80	2.20	fry in pan and stew	0.80 * 0.90	12.80
	Onion	100.00	0.00	0.66	0.40	0.00	fry in pan and stew	0.80 * 0.90	0.00
	Vinegar	4.50	0.00	0.00	0.00	0.00	stew	1.00	0.00
	Flour	7.50	0.00	0.00	0.00	0.00	stew	1.00	0.00
	Pepper	1.25	2.38	2.26	0.00	0.00	stew	1.00	2.38
	Butter	12.50	113.13	103.09	87.50	92.26	stew	1.00	113.13
	Laurel	1.25	0.00	0.03	0.00	0.00	stew	1.00	0.00
	Cloves	1.25	0.63	0.33	0.70	0.00	stew	1.00	0.63
	Bouillon powder	1.25	0.00	0.00	0.00	0.00	stew	1.00	0.00
	Total		136.13	108.34	123.40	94.46			128.93

Table S7. Vitamin B1 calculation for three recipes

stamppot		Raw weight in recipe (g)	VB1 mg in recipe(NEVO)	VB1 mg in recipe (Mynetdiary)	VB1 mg in recipe (Calories!)	VB1 mg in recipe(Virtue)	First and second cooking procedure	First and second retention factor	VB1 mg in one portion (NEVO)
	kale	250.00	0.50	0.25	0.25	0.28	stew	0.90	0.45
	potatoes	312.50	0.13	0.06	0.31	0.22	stew	0.90	0.11
	butter	10.00	0.00	0.00	0.00	0.00	boil	1.00	0.00
	semi-skimmed milk	18.80	0.03	0.00	0.01	0.00	boil	0.90	0.03
	Total		0.66	0.32	0.57	0.49			0.60
Pizza	Flour	50.00	0.10	0.40	0.05	0.40	bake in oven	0.75	0.08
	Olive oil	4.50	0.00	0.00	0.00	0.00	bake in oven	-	0.00
	Tomato puree	11.25	0.02	0.00	0.02	0.00	bake in oven	0.90	0.02
	Yeast	0.88	0.02	0.10	0.01	0.10	bake in oven	-	0.02
	Oregano	0.38	0.00	0.00	0.00	0.00	bake in oven	-	0.00
	Mature cheese 48+	37.50	0.00	0.00	0.01	0.01	bake in oven	0.75	0.00
	Tomato	25.00	0.00	0.00	0.01	0.01	bake in oven	0.90	0.01
	Salami	25.00	0.05	0.23	0.18	0.23	bake in oven	0.90	0.04
	Mushroom	25.00	0.02	0.03	0.02	0.02	bake in oven	0.90	0.02
	Total		0.21	0.75	0.29	0.77			0.18
Hachee	Hachee meat	100.00	0.05	0.10	0.10	0.08	fry in pan and stew	0.60 * 0.60	0.02
	Onion	100.00	0.04	0.00	0.05	0.05	fry in pan and stew	0.90 * 0.90	0.03
	Vinegar	4.50	0.00	-	0.00	0.00	stew	1.00	0.00
	Flour	7.50	0.01	0.08	0.01	0.06	stew	0.75	0.00
	Pepper	1.25	0.00	0.01	0.00	0.00	stew	1.00	0.00
	Butter	12.50	0.00	0.00	0.00	0.00	stew	1.00	0.00
	Laurel	1.25	0.00	0.00	0.00	0.00	stew	1.00	0.00
	Cloves	1.25	0.00	0.00	0.00	0.00	stew	1.00	0.00
	Bouillon powder	1.25	0.00	0.00	0.00	0.00	stew	1.00	0.00
	Total		0.10	0.19	0.16	0.19			0.06

Table S8. Vitamin B2 calculation for three recipes

stamppot		Raw weight in recipe (g)	VB2 mg in recipe(NEVO)	VB2 mg in recipe (Mynetdiary)	VB2 mg in recipe (Calories!)	VB2 mg in recipe(Virtue)	First and second cooking procedure	First and second retention factor	VB2 mg in one portion (NEVO)
	kale	250.00	0.05	0.25	0.75	0.33	stew	0.95	0.05
	potatoes	312.50	0.38	0.31	0.00	0.09	stew	0.95	0.36
	butter	10.00	0.00	0.00	0.01	0.00	boil	1.00	0.00
	semi-skimmed milk	18.80	0.01	0.00	0.03	0.06	boil	0.95	0.01
	Total		0.43	0.56	0.79	0.48			0.41
Pizza	Flour	50.00	0.03	0.25	0.05	0.25	bake in oven	1.00	0.03
	Olive oil	4.50	0.00	0.00	0.00	0.00	bake in oven	-	0.00
	Tomato puree	11.25	0.01	0.01	0.01	0.01	bake in oven	0.95	0.01
	Yeast	0.88	0.04	0.04	0.02	0.04	bake in oven	-	0.04
	Oregano	0.38	0.00	0.00	0.00	0.00	bake in oven	-	0.00
	Mature cheese 48+	37.50	0.11	0.15	0.15	0.14	bake in oven	0.95	0.10
	Tomato	25.00	0.00	0.00	0.00	0.01	bake in oven	0.95	0.00
	Salami	25.00	0.05	0.08	0.05	0.08	bake in oven	1.00	0.05
	Mushroom	25.00	0.08	0.10	0.10	0.10	bake in oven	0.95	0.07
	Total		0.31	0.62	0.38	0.62			0.30
Hachee	Hachee meat	100.00	0.16	0.20	0.20	0.17	fry in pan and stew	1.00 * 1.00	0.16
	Onion	100.00	0.02	0.00	0.03	0.03	fry in pan and stew	0.95 * 0.95	0.02
	Vinegar	4.50	0.00	-	0.00	0.00	stew	1.00	0.00
	Flour	7.50	0.00	0.00	0.00	0.04	stew	1.00	0.00
	Pepper	1.25	0.00	0.00	0.00	0.00	stew	1.00	0.00
	Butter	12.50	0.00	0.00	0.01	0.00	stew	1.00	0.00
	Laurel	1.25	0.00	0.01	0.00	0.01	stew	1.00	0.00
	Cloves	1.25	0.00	0.00	0.00	0.00	stew	1.00	0.00
	Bouillon powder	1.25	0.00	0.00	0.00	0.00	stew	1.00	0.00
	Total		0.19	0.21	0.24	0.25			0.19

Table S9. Vitamin B6 calculation for three recipes

stamppot		Raw weight in recipe (g)	VB6 mg in recipe(NEVO)	VB6 mg in recipe (Mynetdiary)	VB6 mg in recipe (Calories!)	VB6 mg in recipe(Virtue)	First and second cooking procedure	First and second retention factor	VB6 mg in one portion (NEVO)
	kale	250.00	0.55	0.75	0.75	0.68	stew	0.90	0.50
	potatoes	312.50	0.94	0.63	0.94	0.63	stew	0.90	0.84
	butter	10.00	0.00	0.00	0.00	0.00	boil		0.00
	semi-skimmed milk	18.80	0.01	0.00	0.01	0.00	boil	0.80	0.01
	Total		1.49	1.38	1.70	1.30			1.34
Pizza	Flour	50.00	0.13	0.00	0.05	0.02	bake in oven	0.90	0.11
	Olive oil	4.50	0.00	0.00	0.00	0.00	bake in oven	-	0.00
	Tomato puree	11.25	0.03	0.01	0.05	0.01	bake in oven	0.90	0.03
	Yeast	0.88	0.02	0.01	0.01	0.01	bake in oven	-	0.02
	Oregano	0.38	0.00	0.00	0.00	0.00	bake in oven	-	0.00
	Mature cheese 48+	37.50	0.01	0.04	0.04	0.03	bake in oven	0.75	0.01
	Tomato	25.00	0.02	0.03	0.02	0.02	bake in oven	0.90	0.02
	Salami	25.00	0.03	0.15	0.13	0.14	bake in oven	0.90	0.02
	Mushroom	25.00	0.03	0.03	0.03	0.03	bake in oven	0.90	0.03
	Total		0.26	0.27	0.31	0.26			0.24
Hachee	Hachee meat	100.00	0.26	0.60	0.20	0.57	fry in pan and stew	0.60 * 0.60	0.09
	Onion	100.00	0.12	0.10	0.12	0.12	fry in pan and stew	0.90 * 0.90	0.10
	Vinegar	4.50	0.00	-	0.00	0.00	stew	1.00	0.00
	Flour	7.50	0.01	0.00	0.02	0.00	stew	0.80	0.01
	Pepper	1.25	0.00	0.00	0.00	0.00	stew	1.00	0.00
	Butter	12.50	0.00	0.00	0.00	0.00	stew	1.00	0.00
	Laurel	1.25	0.00	0.02	0.00	0.02	stew	1.00	0.00
	Cloves	1.25	0.00	0.01	0.00	0.01	stew	1.00	0.00
	Bouillon powder	1.25	0.00	0.00	0.00	0.00	stew	1.00	0.00
	Total		0.39	0.73	0.34	0.73			0.20

Table S10. Vitamin B12 calculation for three recipes

stamppot		Raw weight in recipe (g)	VB12 ug in recipe(NEVO)	VB12 ug in recipe (Mynetdiary)	VB12 ug in recipe (Calories!)	VB12 ug in recipe(Virtue)	First and second cooking procedure	First and second retention factor	VB12 ug in one portion (NEVO)
	kale	250.00	0.00	0.00	-	0.00	stew	-	0.00
	potatoes	312.50	0.00	0.00	-	0.00	stew	-	0.00
	butter	10.00	0.03	0.02	-	0.02		1.00	0.03
	semi-skimmed milk	18.80	0.08	0.41	-	0.17	boil	0.90	0.08
	Total		0.11	0.43	-	0.19			0.11
Pizza	Flour	50.00	0.00	0.00	-	0.00	bake in oven	1.00	0.00
	Olive oil	4.50	0.00	0.00	-	0.00	bake in oven	-	0.00
	Tomato puree	11.25	0.00	0.00	-	0.00	bake in oven	0.70	0.00
	Yeast	0.88	0.00	0.00	-	0.00	bake in oven	-	0.00
	Oregano	0.38	0.00	0.00	-	0.00	bake in oven	-	0.00
	Mature cheese 48+	37.50	0.75	0.30	-	0.31	bake in oven	0.90	0.68
	Tomato	25.00	0.00	0.00	-	0.00	bake in oven	0.70	0.00
	Salami	25.00	0.35	0.70	-	0.70	bake in oven	0.95	0.33
	Mushroom	25.00	0.00	0.00	-	0.01	bake in oven	0.70	0.00
	Total		1.10	1.00	-	1.02			1.01
Hachee	Hachee meat	100.00	2.91	2.53	-	2.66	fry in pan and stew	0.70 * 0.70	1.43
	Onion	100.00	0.00	0.00	-	0.00	fry in pan and stew	0.70 * 0.70	0.00
	Vinegar	4.50	-	0.00	-	0.00	stew	1.00	0.00
	Flour	7.50	0.00	0.00	-	0.00	stew	0.95	0.00
	Pepper	1.25	0.00	0.00	-	0.00	stew	1.00	0.00
	Butter	12.50	0.04	0.03	-	0.02	stew	1.00	0.04
	Laurel	1.25	0.00	0.00	-	0.00	stew	1.00	0.00
	Cloves	1.25	0.00	0.00	-	0.00	stew	1.00	0.00
	Bouillon powder	1.25	0.00	0.14	-	0.00	stew	1.00	0.00
	Total		2.95	2.70	-	2.69			1.46

Table S11. Folate calculation for three recipes

stamppot		Raw weight in recipe (g)	Folate ug in recipe(NEVO)	Folate ug in recipe (Mynetdiary)	Folate ug in recipe (Calories!)	Folate ug in recipe(Virtue)	First and second cooking procedure	First and second retention factor	Folate ug in one portion (NEVO)
	kale	250.00	125.00	350.00	0.00	0.00	stew	0.70	87.50
	potatoes	312.50	71.88	56.25	93.75	0.00	stew	0.75	53.91
	butter	10.00	0.00	0.30	0.30	0.00		-	0.00
	semi-skimmed milk	18.80	1.22	0.00	0.00	0.00	boil	0.50	0.61
	Total		198.10	406.55	94.05	0.00			142.02
Pizza	Flour	50.00	27.00	91.50	8.00	77.00	bake in oven	0.50	13.50
	Olive oil	4.50	0.00	0.00	0.00	0.00	bake in oven	-	0.00
	Tomato puree	11.25	4.64	1.24	3.68	0.00	bake in oven	0.70	3.24
	Yeast	0.88	35.00	20.48	8.22	0.00	bake in oven	-	35.00
	Oregano	0.38	0.00	0.89	0.00	0.00	bake in oven	-	0.00
	Mature cheese 48+	37.50	9.38	6.75	12.38	0.00	bake in oven	0.50	4.69
	Tomato	25.00	3.93	3.75	9.75	0.00	bake in oven	0.70	2.75
	Salami	25.00	0.63	0.50	0.50	0.00	bake in oven	0.80	0.50
	Mushroom	25.00	11.00	4.25	2.00	0.00	bake in oven	0.70	7.70
	Total		91.56	129.35	44.52	77.00			67.38
Hachee	Hachee meat	100.00	2.90	3.30	13.00	0.00	fry in pan and stew	0.80 * 0.80	1.86
	Onion	100.00	23.70	20.90	14.60	0.00	fry in pan and stew	0.70 * 0.70	11.61
	Vinegar	4.50	0.00	0.00	0.00	0.00	stew	1.00	0.00
	Flour	7.50	1.43	28.60	0.80	12.71	stew	0.80	1.14
	Pepper	1.25	0.00	0.23	0.00	0.00	stew	1.00	0.00
	Butter	12.50	0.00	0.41	0.40	0.00	stew	1.00	0.00
	Laurel	1.25	0.00	2.48	0.00	0.00	stew	1.00	0.00
	Cloves	1.25	0.00	0.34	0.00	0.00	stew	1.00	0.00
	Bouillon powder	1.25	0.00	0.44	0.00	0.00	stew	1.00	0.00
	Total		28.03	56.70	28.80	12.71			14.61
